# One-Year Outcomes of Trabeculotomy with 120°, 180°, or 360° Schlemm’s Canal Incision for Primary Open-Angle Glaucoma: A Retrospective Study

**DOI:** 10.3390/jcm13247653

**Published:** 2024-12-16

**Authors:** Hidetsugu Mori, Tatsunori Kiriishi, Masatoshi Omi, Masayuki Ohnaka, Hisanori Imai

**Affiliations:** Department of Ophthalmology, Kansai Medical University, Hirakata 573-1010, Osaka, Japan

**Keywords:** primary open-angle glaucoma, trabeculotomy, ab interno, ab externo, metal probe, Kahook Dual Blade

## Abstract

**Background/Objectives:** Primary open-angle glaucoma (POAG), if caused by elevated intraocular pressure (IOP), may require a trabeculotomy (LOT), in which the trabecular meshwork (TM) and Schlemm’s canal (ISC) are incised. However, the association between the incision angle and outcomes remains unclear. Therefore, in this study, we investigated the surgical outcomes of a trabeculotomy combined with cataract surgery in patients with POAG over a 12-month follow-up period. **Methods:** We included 66 patients (corresponding to 83 eyes) with POAG who underwent trabeculotomy ab externo with a metal probe (M-LOT: 120° incision of the TM and ISC), ab interno with a Kahook Dual Blade^®^ (K-LOT: 180° incision of the TM and ISC), or ab interno with a 5-0 nylon suture (S-LOT: 360° incision of the TM and ISC) between January 2015 and December 2022. We assessed IOP, the percentage reduction from preoperative IOP, the number of IOP-lowering medications taken, the incidence of postoperative complications, and the success rate using Kaplan–Meier survival analysis. **Results:** The median IOP was significantly lower than the baseline across all three groups. The number of IOP-lowering medications taken was significantly reduced only in the M-LOT group. The mean percentage reduction from the preoperative IOP in the K-LOT group was significantly lower than that in the M and S-LOT groups. Beween those subjected to an ab ineterno LOT, the S-LOT group demonstrated a significantly higher rate of IOP reduction 12 months after the operation compared to the K-LOT group. Kaplan–Meier cumulative survival analyses revealed a lower success rate for the K-LOT group than for the M and S-LOT groups. The M-LOT group had the lowest incidence of hyphema and IOP spikes, whereas the S-LOT group had the highest incidence of these complications. **Conclusions:** The M-, K-, and S-LOTs had different surgical outcomes during the 12 months of follow-up, with the M-LOT group showing the fewest complications. These results will help in selecting the most suitable trabeculotomy strategy for patients with POAG. Based on the postoperative outcomes of the ab interno K- and S-LOTs, a wider incision of the TM and ISC leads to effective IOP reduction.

## 1. Introduction

Glaucoma is a progressive disease and the second leading cause of irreversible visual blindness worldwide. It is characterized by functional and structural changes in the optic nerve, typically associated with retinal ganglion cell death [1]. Elevated intraocular pressure (IOP) is a major risk factor for glaucoma occurrence and progression [2,3]. Maintaining a low IOP is thus considered the main treatment strategy for preventing both visual loss and the progression of visual field defects in patients with glaucoma.

The aqueous humor is produced in the nonpigmented portion of the ciliary body and drains through two pathways: the conventional outflow pathway via the trabecular meshwork (TM) and the uveoscleral pathway through the ciliary body to the suprachoroidal space. The main barriers to aqueous humor outflow in glaucomatous eyes are the TM and the inner walls of the Schlemm’s canal (ISC) [4,5].

Topical treatments, including medical or laser treatments, are usually selected as the first-line therapy for patients with glaucoma. However, if topical treatments fail to sufficiently reduce IOP, a second-line therapy with a trabeculotomy may be used to relieve aqueous outflow resistance from the anterior chamber to the collector channels via surgically opening the walls of the TM and ISC, thus reducing IOP without bleb formation. Conventional trabeculotomy is performed using metal trabecular probes in an ab externo approach, which involves extensive conjunctival and scleral incisions and sutures, as in a trabeculectomy [6,7]. Since the 2012 introduction of the ab interno approach in minimally invasive glaucoma surgery (MIGS) [8], new devices have been widely adopted. Common MIGS techniques include gonioscopy-assisted transluminal trabeculotomies with equipment such as the Kahook Dual Blade (KDB; New World Medical, Rancho Cucamonga, CA, USA) [9], iStent (Glaukos, Laguna Hills, CA, USA) [10], Trabectome (NeoMedix, Tustin, CA, USA) [11], Microhook [12], and 5-0 nylon sutures [13]. MIGS, an ab interno trabeculotomy procedure, has emerged as an alternative to conventional ab externo trabeculotomy approaches for glaucoma because of the relative ease of this surgical procedure, its short surgical time, and its ability to preserve the conjunctiva and sclera. Glaucoma surgeons must select the appropriate procedure based on a thorough understanding of the potential postoperative outcomes of each procedure.

Among the various types of glaucoma, primary open-angle glaucoma (POAG) is the most common worldwide, with increasing incidence in older populations [14,15]. Furthermore, a potentially large subset of patients require treatment for both glaucoma and coexisting mild senile cataracts. Several previous studies [16,17,18,19,20] have assessed the clinical use of ab interno and ab externo trabeculotomies with cataract surgery; however, these reports were lacking in some aspects. First, the patients were not classified according to the type of open-angle glaucoma, such as POAG, normal-tension glaucoma (NTG), and exfoliation glaucoma. Second, assessments of the surgical outcomes did not distinguish between trabeculotomy alone and trabeculotomy with cataract extraction. Third, the surgical techniques used for traditional ab externo trabeculotomy differed among the glaucoma surgeons. A traditional ab externo trabeculotomy involves either the incision of the TM and ISC only or this procedure combined with the removal of Schlemm’s canal endothelium and deep sclerectomy (SER + DS). Notably, few prior reports, including our own [19,20], have assessed conventional ab externo trabeculotomy with SER and DS.

To select the appropriate trabeculotomy strategy among the various options with an ab interno-externo approach, glaucoma surgeons should understand the potential surgical outcomes of each procedure.

In this retrospective 12-month follow-up study, we compared the efficacy and complication rates of traditional ab externo 120° trabeculotomy with those of SER + DS using a metal probe, ab interno 180° trabeculotomy using the KDB, and ab interno 360° trabeculotomy using 5-0 nylon sutures, all in combination with cataract surgery, in patients with POAG (excluding NTG).

## 2. Materials and Methods

We enrolled patients who underwent ab interno or ab externo trabeculotomy with cataract surgery at the Kansai Medical University Hospital, Japan, between January 2015 and December 2022. This study was conducted in accordance with the guidelines of the Declaration of Helsinki and approved by the Institutional Review Board of Kansai Medical University through a review of our electronic medical records. Informed consent was obtained from all patients. All patients underwent complete ophthalmic examinations, including best-corrected visual acuity, slit-lamp biomicroscopy, gonioscopy, fundoscopy, and IOP measurements using Goldmann applanation tonometry.

POAG was diagnosed based on an IOP > 21 mmHg with or without IOP-lowering medications, open-angle glaucoma (Shaffer grade 3 or 4) determined using gonioscopy, and optic neuropathy with matching optic disc and visual field defects. Patients with other pre- or co-existing ocular diseases, those with a history of steroid therapy, those with ocular trauma, and those with prior ocular surgery, including laser treatment, narrow-angle glaucoma determined via gonioscopy, and complications during surgery, were excluded. Patients with POAG who were followed up for <12 months were also excluded from the analysis. Cataract surgery was indicated if the patient complained of glare or halos, had a nuclear grade ≥ 2 (Emery-Little grading), or had a best-corrected visual acuity of ≤20/20. Patients with POAG and cataracts were recruited from the hospital’s electronic medical records.

All surgeries were performed under local anesthesia by experienced glaucoma specialists (first author and co-authors).

### 2.1. Surgical Techniques

Trabeculotomy via an Ab Externo Approach Using a Metal Trabecular Probe (M-LOT)

After incising the conjunctiva and sub-Tenon tissue in the inferior nasal or temporal area, a 4 × 4 mm superficial scleral flap was made, followed by a similarly sized deep scleral flap. Corneal side ports were created at the 3 and 9 o’clock positions using a 20-gauge (G) V lance, and a continuous curvilinear capsulorhexis (CCC) was then performed, with the anterior chamber filled with ophthalmic viscosurgical devices (OVDs). An inferior nasal or temporal 120° incision of the TM and ISC was then performed using a metal trabecular probe. The second scleral flap was then removed (SER and DS), and the first scleral flap and conjunctiva were then sutured using 10-0 nylon.

Trabeculotomy via an Ab Interno Approach Using a Kahook Dual Blade^®^ (K-LOT)

After creating a corneal side port at the 9 o’clock position using a 20 G V lance, the anterior chamber was filled with OVDs, and CCC was completed. The angle was visualized using an Ocular Swan Jacob Gonioprism (Ocular Instruments, Bellevue, WA, USA) by tilting both the patient’s head and the microscope. The tip of the KDB was then inserted into Schlemm’s canal through the TM and moved circumferentially to excise the TM and ISC from 6 to 12 o’clock (nasal bisection: 180°).

Trabeculotomy via an Ab Interno Approach Using a 5-0 Nylon Suture (S-LOT)

Corneal side ports were created at the 7 and 12 o’clock positions using a 20 G V lance. After filling the anterior chamber with OVDs, a CCC was performed. A suture was then inserted into the anterior chamber through a corneal side port at 7 o’clock. Using a Swan-Jacob Gonioprism and/or Mori Upright Surgical Gonio Lens (Ocular Instruments, Bellevue, WA, USA), an approximately 1–1.5 mm incision of the TM was made through the corneal side port at 12 o’clock using a 20 G V lance. The tip of a 5-0 nylon (Mani Nylon, Mani, Tochigi, Japan) suture was inserted into Schlemm’s canal through the TM and advanced circumferentially along the ISC using 23-G disposable microsurgical forceps (DSP forceps, Alcon, Tokyo, Japan). After passing the suture tip around the circumference of Schlemm’s canal, the distal edge of the suture was pulled out from the same opening, completing a 360° incision of the TM and ISC.

Phacoemulsification

### 2.2. Parameters Evaluated

We investigated the following parameters: sex, age, IOP, percentage reduction from preoperative to final IOP, the number of IOP-lowering medications employed, and the incidence rate of early postoperative complications within 1 month. The baseline IOP was determined as the mean of the three most recent measurements taken prior to trabeculotomy. We employed prostaglandin, beta-blocker, carbonic anhydrase inhibitors, alpha2-agonits, and Rock inhibitors as anti-glaucoma medications. The use of a combination of IOP-lowering medications was counted as two separate drugs. The most frequent early postoperative complications (<1 month after surgery) included hyphema with niveau formation with > 1 mm of blood in the anterior chamber detected using slit-lamp biomicroscopy, and an IOP spike, defined as a transient elevation of >30 mmHg. Postoperative data on IOP and the number of IOP-lowering medications were collected at 1 week, and then at 1, 3, 6, 9, and 12 months postoperatively.

### 2.3. Statistical Analyses

All statistical analyses were conducted using JMP software version 15 (SAS Inc., Cary, NC, USA). Values are presented as the mean ± standard deviation for normally distributed data and as the median and interquartile range for non-normally distributed data. Dunn’s multiple comparison tests were used to compare pre- and postoperative IOP values. The Tukey–Kramer test was used to compare the rate of IOP reduction from baseline. Dunnet’s test was used to compare the number of IOP-lowering medications employed pre- and postoperatively. Kaplan–Meier survival curve analysis was used to evaluate the cumulative probability of success, and the M-, K-, and S-LOT procedures were compared using the log-rank test. We defined surgical failure as < 20% reduction from the preoperative IOP value, a postoperative IOP > 21 or <5 mmHg on two consecutive visits, and additional glaucoma surgery at least 1 month after the initial surgery. Statistical significance was set at *p* value < 0.05.

## 3. Results

A total of 83 eyes from 66 patients were included. M-, K-, and S-LOTs with PEA + IOLs were performed on 35, 36, and 12 eyes, respectively. Table 1 summarizes the clinical characteristics of the patients.

**Figure 1:**
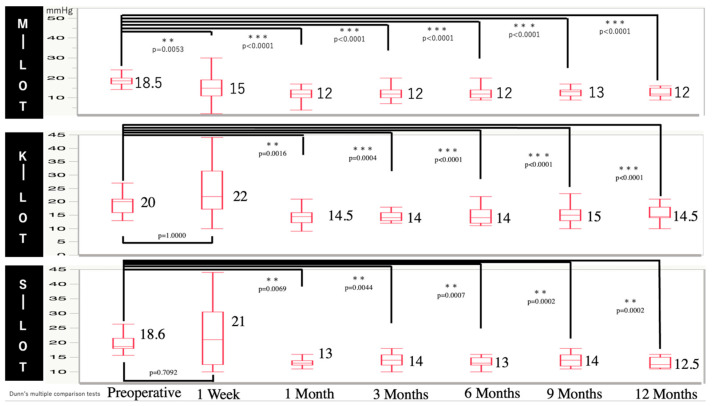
Time course of IOP in patients with POAG receiving M-, K-, and S-LOTs.

Figure 1 shows the baseline and postoperative IOP values at each time point for all the groups. Postoperative IOP was significantly reduced starting from 1 week to 12 months compared to the baseline in all groups.

**Figure 2:**
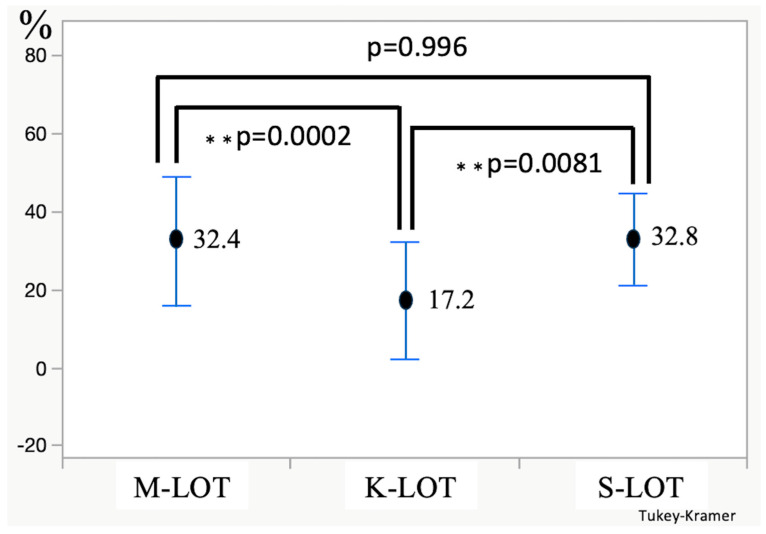
The rate of IOP reduction from before the operation to 12 months after the operation.

As shown in Figure 2, the percentage of IOP reduction from before the operation to 12 months after was 32.4% for M-LOT, 17.2% for K-LOT, and 32.8% for S-LOT. The K-LOT group demonstrated a significantly lower percentage of IOP reduction than the M- and S-LOT groups (K vs. M-LOT, *p* = 0.0002; K vs. S-LOT, *p* = 0.0081; M vs. S-LOT, *p* = 0.996).

**Figure 3:**
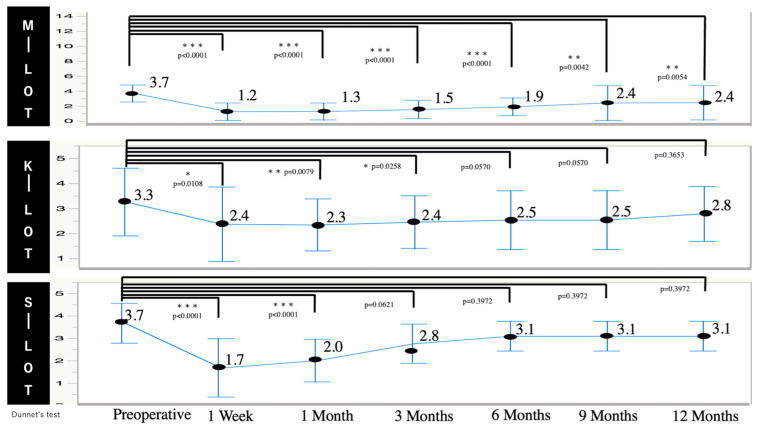
Time course of changes in the number of IOP-lowering medications for patients with POAG who were subjected to M-, K-, and S-LOTs.

Figure 3 shows the changes in the mean number of IOP-lowering medications employed before and after surgery for patients with POAG. The mean number of IOP-lowering medications employed for the POAG patients in the M-LOT group decreased significantly from 3.7 ± 1.1 at the baseline to 1.2 ± 1.2 at 1 week (*p* < 0.0001) and 2.4 ± 2.3 at 12 months (*p* = 0.0054) postoperatively (Figure 3). For the K-LOT group, the number of IOP-lowering medications was significantly reduced at 1 week, 1 month, and 3 months (2.4 ± 1.5 vs. 2.3 ± 1.0 vs. 2.4 ± 1.1, respectively) compared to the baseline (3.3 ± 1.3). For the S-LOT group, the number of IOP-lowering medications was significantly reduced at 1 week and 1 month (1.7 ± 1.3 vs. 2.0 ± 1.0, respectively) compared to the baseline (3.7 ± 0.9).

**Figure 4:**
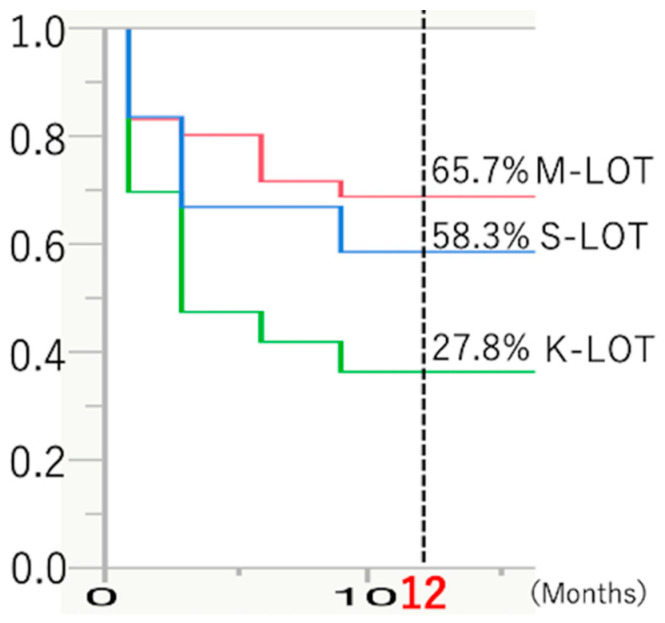
Kaplan–Meier survival curves for patients treated with M-, K-, or S-LOTs with cataract surgery.

The Kaplan–Meier cumulative survival curves for the patients with POAG in the three treatment groups (M-, K-, and S-LOT) are presented in Figure 4. The success rate for K-LOT (27.8%) was significantly lower than that for M- and S-LOTs for patients with POAG (65.7%, 58.3%) 12 months postoperatively.

Table 2 presents the occurrence of early postoperative complications across all groups. Fortunately, the hyphema and transient IOP spikes regressed spontaneously within 1–2 weeks without anterior chamber washing in all cases. Among patients with POAG, hyphema and transient IOP spikes were less frequent in the M-LOT group than in the S- and K-LOT groups.

## 4. Discussion

In this retrospective study, we evaluated and compared the surgical outcomes of 360° trabeculotomy ab interno with sutures, nasal 180° trabeculotomy ab interno with KDB, and 120° trabeculotomy ab externo with a metal probe in combination with cataract extraction during 12 months of postoperative follow-ups for patients with POAG.

Whether the reduction in IOP is correlated with the extent of the TM and ISC incisions remains unclear. Postoperative efficacy related to the incision range in LOT procedures in previous procedures varies. Manabe et al. [21] reported that an incision of ≥150° in the TM and ISC did not significantly affect IOP reduction compared with the 1-year outcome of an S-LOT via an ab externo approach. Sato et al. [16] also showed that neither a 180° nor a 360° incision in the TM and ISC resulted in a significant IOP reduction. In enucleated human eyes, 120° trabeculotomy caused an 85% IOP reduction in outflow resistance [22]. These prior experimental and clinical results suggest that incisions >120–180° may not contribute significantly to IOP reduction [16,21,22]. In contrast, Chin et al. reported that a 360° suture trabeculotomy achieved lower IOP values than a 120° metal trabeculotomy [13]. However, evaluating surgical outcomes based on the extent of the TM and ISC incisions is challenging because of the different incision approaches used. Our current study demonstrates that the patients in the M-LOT group, with the smallest (120°) TM and ISC incisions, had the lowest postoperative IOP values when compared with the K- and S-LOT groups, showing an approximately 30% reduction in IOP, similar to the S-LOT with 360° TM and ISC incisions. In contrast, the K-LOT group with 180° TM and ISC incisions showed the lowest percentage of IOP reduction compared to the M- and S-LOT groups. Yasuda et al. [19] reported that an M-LOT with SER + DS sustained a long-term IOP reduction with respect to the baseline. Even after SER + DS is performed, the Schlemm’s canal endothelium should repair itself, leading to the closure of the connection between the anterior chamber and Schlemm’s canal within the first month postoperatively. Our cases showed no bleb formation, as seen in the postoperative trabeculectomy results. Therefore, the filtration effects of SER and DS likely did not contribute to the low rate of postoperative complications in the M-LOT group, consistent with the findings of Yasuda et al. [19]. The mechanism of the long-term IOP reduction in M-LOTs with SER + DS remains unknown. The correlation between the extent of the TM/ISC incision and IOP reduction should be compared between the K- and S-LOT groups, excluding the M-LOT group from this analysis. Wider TM and ISC openings may contribute to greater IOP reduction, as evidenced by the better outcomes associated with the 360° incision in the S-LOT group than those associated with the 180° incision in the K-LOT group. Our current study demonstrates that the extent of TM and ISC incisions might correlate with the reduction in IOP or the number of IOP-lowering medications in the S-LOT group compared to the K-LOT group.

Hyphema with niveau formation and transient IOP spikes are the most frequent early complications associated with trabeculotomy. Hyphema with niveau formation results from blood reflux from the episcleral veins after a TM incision in the Schlemm’s canal, while transient IOP spikes are related to prolonged hyphema with niveau formation in the anterior chamber. In the present study, the incidence rates of both hyphema with niveau formation and transient IOP spikes were lowest in the M-LOT group. Shorter incisions in the TM and ISC may reduce blood reflux, leading to a lower frequency of postoperative hyphema with niveau formation and transient IOP spikes. Moreover, the filtration effects of SER and DS within 1 month after the operation may have contributed to the low rate of postoperative complications in the M-LOT group, consistent with the findings reported by Yasuda E et al. [19].

In our study, the M-LOT with an ab externo approach was associated with the lowest postoperative median IOP at final presentation, a higher rate of IOP reduction, the lowest number of IOP-lowering medications, the highest rate of survival in the Kaplan–Meier survival analysis, and the lowest rates of hyphema with niveau formation and transient IOP spikes during the 12 months of postoperative follow-up. Our study suggests that the conventional M-LOT with an ab externo approach may be more effective than the S-LOT or K-LOT with an ab interno approach for patients with POAG.

The findings of this study suggest that the optimal trabeculotomy strategy should be selected based on specific criteria for each patient. For individuals with POAG, the choice between M-, K-, or S-LOT should be guided by the target postoperative IOP. For target IOPs of 12–13 mmHg, M-LOT is recommended; for 13–14 mmHg, S-LOT is preferred; and for values above this range, K-LOT may be used. When choosing the M-LOT, glaucoma surgeons should keep in mind that this procedure requires conjunctival and scleral dissection because it is performed using the ab externo approach. The main advantage of ab interno approaches, other than M-LOT, is that they do not require conjunctival or scleral dissection and allow for subsequent filtration surgeries, if necessary. M-LOT, featuring conjunctival and scleral dissection, should be selected for patients who do not require filtering devices, including Baerveldt^®^, Ahmed^®^, Express^®^, and so on. M-LOT may also be selected to avoid transient IOP spikes and/or worsening of visual acuity due to hyphema with niveau formation, especially in cases of severe glaucoma or if the condition affects the dominant eye. Conversely, K-LOT can be selected if one expects that a second ab interno trabeculotomy needs to be conducted due to the presence of residual 180° incisions in the temporal area of the TM and ISC. Although trabeculectomy should initially be performed based on the severity of POAG, S-LOTs may be preferred because of difficulties in postoperative management, especially for patients with dementia or poor ADL due to other systemic diseases.

Our study has several limitations. First, this was a retrospective study, and the 120°, 180°, and 360° TM and ISC incisions were therefore not randomly assigned to the participants. Future prospective studies with randomized assignment of incision types could provide more robust data. The decision to discontinue medication was based on the investigator’s discretion rather than a pre-specified protocol. However, selection bias was limited by selecting all cases that met the minimal eligibility criteria within the specified timeframe. Second, the patients were recruited regardless of glaucoma severity. Given that severe glaucoma may affect surgical outcomes, differences in severity among the three groups may have affected the study results. Third, cataract surgery reduces IOP via several mechanisms. Increased postoperative outflow can result from anatomical changes, such as angle width or the traction of the ciliary zonules in pseudophakic eyes after cataract surgery. Notably, our study population included patients who underwent combined trabeculotomy and cataract surgery.

## 5. Conclusions

In conclusion, the goal of glaucoma management is to preserve a patient’s quality of life by maintaining their visual field and visual acuity and minimize any adverse effects of the treatment. Differences in the extent and location of Schlemm’s canal incisions during ab interno and/or ab externo trabeculotomy, including 360°, 180°, and 120° incisions, resulted in good surgical outcomes during the 12-month follow-up period for patients with POAG. To our knowledge, no previous reports have compared the range of postoperative results for trabeculotomies using the ab interno and externo approaches, as described in the present study. Although we are entering the era of MIGS for patients with glaucoma, the M-LOT should remain a highly utilized surgical option. A better understanding of the outcomes of the different procedures will allow the selection of optimal trabeculotomy strategies for patients with POAG in the future.

## Figures and Tables

**Table 1:** Patient characteristics.

Trabeculotomy (LOT); the Degree of Incision of TM and ISC	POAG (Eyes)
M-LOT; 120°	35
K-LOT; 180°	36
S-LOT; 360°	12

**Table 2:** Early postoperative complications.

POAG	The Rate of IOP-Spike	The Rate of Hyphema
M-LOT	8.6% (3 eyes/35 eyes)	11.4% (4 eyes/35 eyes)
K-LOT	27.8% (10 eyes/36 eyes)	11.1% (4 eyes/36 eyes)
S-LOT	25.0% (3 eyes/12 eyes)	33.3% (4 eyes/12 eyes)

## Data Availability

The authors confirm that the data supporting the findings of this study are available within the article.

## References

[1] Quigley H.A., Broman A.T. (2006). The number of people with glaucoma worldwide in 2010 and 2020. Br. J. Ophthalmol..

[2] Chauhan B.C., Mikelberg F.S., Balaszi A.G., LeBlanc R.P., Lesk M.R., Trope G.E., Canadian Glaucoma Study Group (2008). Canadian Glaucoma Study: 2. Risk factors for the progression of open-angle glaucoma. Arch. Ophthalmol..

[3] Heijl A., Leske M.C., Bengtsson B., Hyman L., Bengtsson B., Hussein M., Early Manifest Glaucoma Trial Group (2002). Reduction of intraocular pressure and glaucoma progression: Results from the Early Manifest Glaucoma Trial. Arch. Ophthalmol..

[4] Johnson M. (2006). What controls aqueous humour outflow resistance?. Exp. Eye Res..

[5] Tamm E.R. (2009). The trabecular meshwork outflow pathways: Structural and functional aspects. Exp. Eye Res..

[6] Tanihara H., Negi A., Akimoto M., Terauchi H., Okudaira A., Kozaki J., Takeuchi A., Nagata M. (1993). Surgical effects of trabeculotomy ab externo on adult eyes with primary open angle glaucoma and pseudoexfoliation syndrome. Arch. Ophthalmol..

[7] Chihara E., Nishida A., Kodo M., Yoshimura N., Matsumura M., Yamamoto M., Tsukada T. (1993). Trabeculotomy ab externo: An alternative treatment in adult patients with primary open-angle glaucoma. Ophthalmic Surg..

[8] Saheb H., Ahmed I.I. (2012). Micro-invasive glaucoma surgery: Current perspectives and future directions. Curr. Opin. Ophthalmol..

[9] Seibold L.K., Soohoo J.R., Ammar D.A., Kahook M.Y. (2013). Preclinical investigation of ab interno trabeculectomy using a novel dual-blade device. Am. J. Ophthalmol..

[10] Samuelson T.W., Katz L.J., Wells J.M., Duh Y.J., Giamporcaro J.E., US iStent Study Group (2011). Randomized evaluation of the trabecular micro-bypass stent with phacoemulsification in patients with glaucoma and cataract. Ophthalmology.

[11] Minckler D.S., Baerveldt G., Alfaro M.R., Francis B.A. (2005). Clinical results with the Trabectome for treatment of open-angle glaucoma. Ophthalmology.

[12] Tanito M., Sano I., Ikeda Y., Fujihara E. (2016). Microhook ab interno trabeculotomy, a novel minimally invasive glaucoma surgery, in eyes with open-angle glaucoma with scleral thinning. Acta Ophthalmol..

[13] Chin S., Nitta T., Shinmei Y., Aoyagi M., Nitta A., Ohno S., Ishida S., Yoshida K. (2012). Reduction of intraocular pressure using a modified 360-degree suture trabeculotomy technique in primary and secondary open-angle glaucoma: A pilot study. J. Glaucoma.

[14] Aboobakar I.F., Johnson W.M., Stamer W.D., Hauser M.A., Allingham R.R. (2017). Major review: Exfoliation syndrome; advances in disease genetics, molecular biology, and epidemiology. Exp. Eye Res..

[15] Iwase A., Suzuki Y., Araie M., Yamamoto T., Abe H., Shirato S., Kuwayama Y., Mishima H.K., Shimizu H., Tomita G. (2004). The prevalence of primary open-angle glaucoma in Japanese: The Tajimi Study. Ophthalmology.

[16] Sato T., Kawaji T. (2021). 12-month randomised trial of 360° and 180° Schlemm’s canal incisions in suture trabeculotomy ab interno for open-angle glaucoma. Br. J. Ophthalmol..

[17] Okada N., Hirooka K., Onoe H., Murakami Y., Okumichi H., Kiuchi Y. (2021). Comparison of efficacy between 120° and 180° Schlemm’s canal incision microhook ab interno trabeculotomy. J. Clin. Med..

[18] Iwasaki K., Takamura Y., Orii Y., Arimura S., Inatani M. (2020). Performances of glaucoma operations with Kahook dual Blade or iStent combined with phacoemulsification in Japanese open angle glaucoma patients. Int. J. Ophthalmol..

[19] Yasuda E., Kanamori A., Ueda K., Akashi A., Sakamoto M., Inoue Y., Yamada Y., Nakamura M. (2016). Trabeculotomy with Schlemm’s canal endothelium removal and deep sclerectomy. Nippon. Ganka Gakkai Zasshi.

[20] Nambu H., Jo N., Kuro M., Minamino K., Ando A., Nambu R., Matsumura M., Takahashi K. (2012). Long-term surgical results of initial trabeculotomy combined with sinusotomy performed inferiorly. Nippon. Ganka Gakkai Zasshi.

[21] Manabe S.I., Sawaguchi S., Hayashi K. (2017). The effect of the extent of the incision in the Schlemm canal on the surgical outcomes of suture trabeculotomy for open-angle glaucoma. Jpn. J. Ophthalmol..

[22] Rosenquist R., Epstein D., Melamed S., Johnson M., Grant W.M. (1989). Outflow resistance of enucleated human eyes at two different perfusion pressures and different extents of trabeculotomy. Curr. Eye Res..

